# Supplementary material for: Four-Chlorophenoxyacetic Acid Treatment Induces the Defense Resistance of Rice to White-Backed Planthopper Sogatella furcifera
Source: Int J Mol Sci. 2023 Oct 29;24(21):15722. doi: 10.3390/ijms242115722 (PMC10648403; doi:10.3390/ijms242115722)
Supplement: Supplementary file 1 [file ijms-24-15722-s001.zip › ijms-2546884-supplementary.pdf]

Supporting Information

Materials and Methods

Quantification of 4-CPA and 4-CP in plants. The HPLC separation was performed on an Agilent ZORBAX C18 column (150 × 2.1 mm, 3.5μm) at 35 °C. The injected volume of sample was 10 μl. The gradient mobile phase consisted of water (solvent A) and acetonitrile (solvent B) at a constant flow rate of 0.3 ml min^{-1}. A linear gradient profile with the following proportions (v/v) of solvent B was applied: 0 to 7 min, 20% to 95% of B; 7 to 10 min, 95% of B. The mass spectrometric analysis was performed using an ESI source in positive ion mode, and the quantification was obtained using multiple reaction monitoring (MRM) mode. The conditions for MS analysis were listed below: capillary voltage of 3,500 V, gas temperature of 325°C, gas flow of 5 L min^{-1}, nebulizing pressure of 45 psi, sheath gas temperature of 350°C, sheath gas flow of 11 L min^{-1} and nozzle voltage of 500 V. The m/z (parent and product) of 4-CPA and 4-CP were 185/127 and 127/91.

POD analysis. Each sample was ground in liquid nitrogen and weighed 100mg. One mL of 0.2 M phosphate buffer (PBS, pH = 7.0) was added to each sample. Samples were vortexed for 5 min and centrifuged (18,000 ×g) for 15 min at 4 °C, and the supernatants were collected. PBS, H_2O_2 and guaiacol were added to a centrifuge tube according to the Peroxidase Activity Assay Kit (Sangon Biotech, #D799592, Shanghai, China). Then supernatant was added to start the oxidation of guaiacol. Record the absorbance value after 30 s and the absorbance value after 1 min 30 s at 470 nm. Enzyme activity was calculated according to the instructions.

H_2O_2 analysis. Each sample was ground in liquid nitrogen and weighed 100mg. One mL of pre-cooled acetone was added to each sample. Samples were vortexed for 5 min and centrifuged (18,000 ×g) for 10 min at 4 °C, and the supernatants were collected. Add lye and titanium sulphate solution to diluted supernatants according to Hydrogen Peroxide Content Assay Kit (Sangon Biotech, #D799774, Shanghai, China). The mixture was centrifuged (4,000 ×g) for 10 min at room temperature and the pellet was collected. Then acid liquor was added to dissolve the pellet. Record the absorbance value of the solution at 415 nm.

Flavonoid and lignin analysis. Each sample was ground in liquid nitrogen and weighed 100 mg for flavonoid analysis and 300 mg for liginin analysis. For flavonoid extraction, each sample was

extracted with 1 mL of 80% aqueous methanol by vortexing for 5 min, followed by ultrasonic treatment for 20 min and finally centrifuged at 18,000 g for 20 min at 4℃. The supernatant was used for flavonoid analysis by HPLC-MS-MS. For lignin extraction, the frozen sample was ground into a powder and homogenized in 5 ml of washing buffer (100 mM K_2HPO_4/KH_2PO_4, 0.5% Triton X-100, 0.5% polyvinylpolypyrrolidone, pH 7.8). The mixture was cultured on a shaker at room temperature at 200 rpm for 30 min, and then centrifuged (6,000 g, 25 °C) for 20 min. The pellet was suspended and washed twice in washing buffer as above and then four times in 100% methanol. The pellet was dried at 80 °C in an oven overnight. The dried pellet was used for lignin analysis with thioglycolic acid.

Artificial diet and rearing procedure. There are four main nutrients - amino acids, vitamins, inorganic salts, sucrose and distilled water - in the pure artificial diet for WBPH. To prepare the diet, all amino acids, vitamins (except ascorbic acid) and trace metals (except $FeCl_3 \cdot 6H_2O$) were prepared as stock solutions of 2×, 10× and 100×, respectively. KH_2PO_4, $MgCl_2 \cdot 6H_2O$, $FeCl_3 \cdot 6H_2O$, amino acid stock, and sucrose were mixed and stirred. Vitamins and trace metal stocks and ascorbic acid solution were then added. The mixture was adjusted to pH 6.8 with 4% KOH. Finally, the solution was filtered through a 0.22 μm filtration membrane to prolong the preservation time. Prepared artificial diets were portioned and stored at – 20 °C and not kept for too long to prevent spoilage. Glass tube (8 cm in height and 4 cm in diameter) were used as feeding chambers. The ends of the glass tubes were sealed with a double layer of parafilms, and 30 μl of artificial diet for WBPH was placed in the middle of the two layers. Change parafilms and artificial diet once a day.

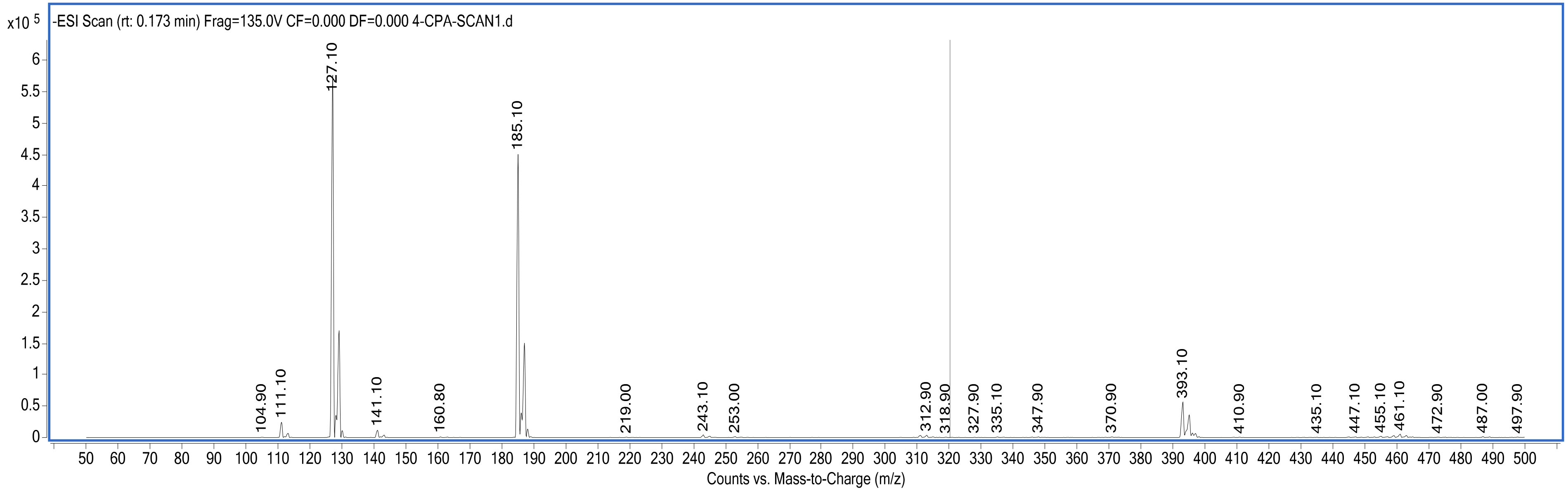

Figure S1. Mass spectra image of 4-CPA.

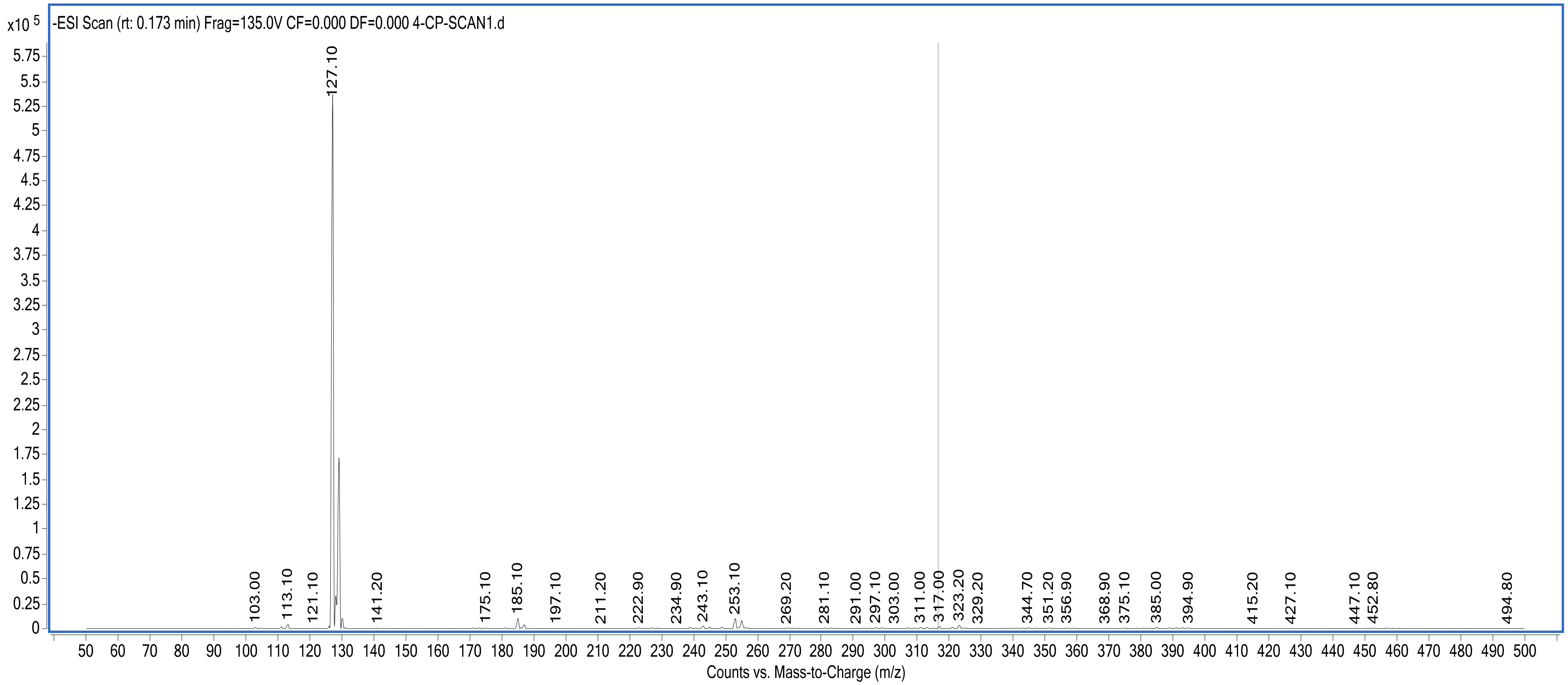

Figure S2. Mass spectra image of 4-CP.

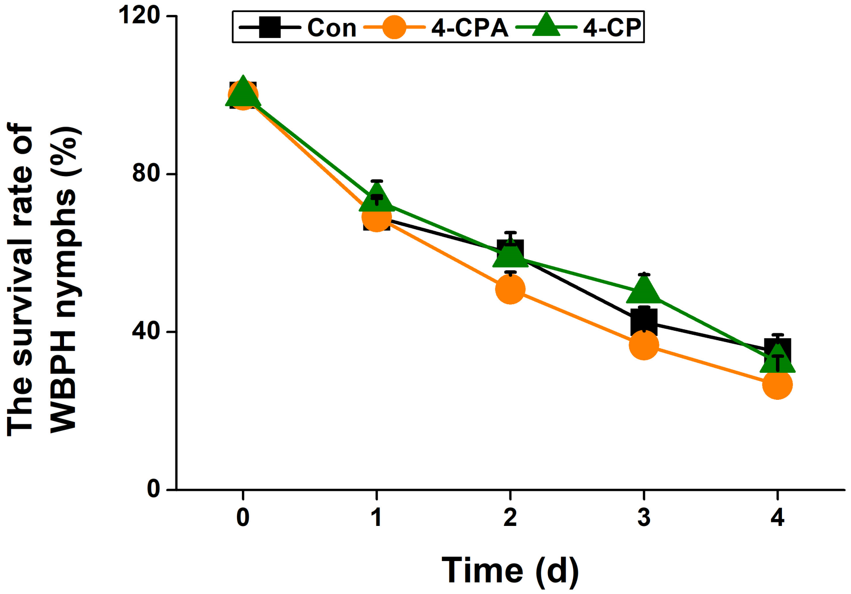

Figure S3. Four-CPA and 4-CP have no direct toxicity to WBPH nymphs. Mean survival rate (+SE, *n* = 8) of 15 newly-hatched WBPH nymphs fed on artificial diet supplemented with 4-CPA or 4-CP; 1 to 4 days after supplement.

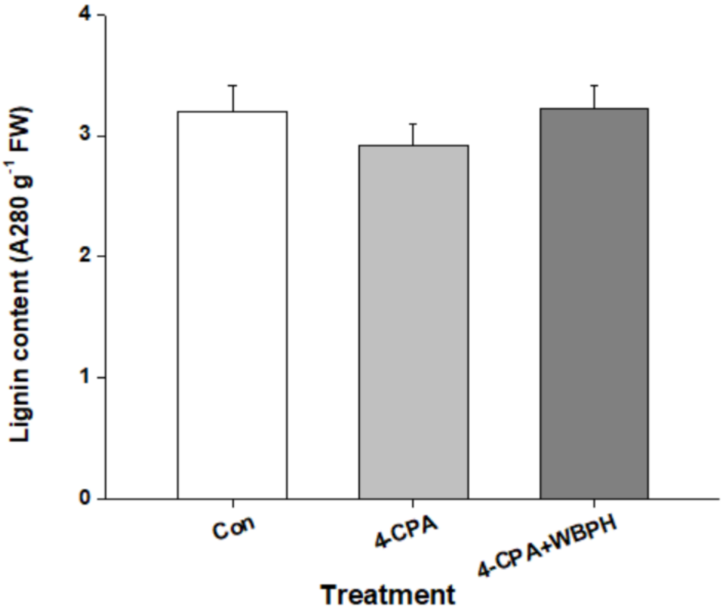

Figure S4. Four-CPA treatment has no effect on lignin levels in rice. Mean levels (+SE, *n*=5) of lignin in plants at 4 d post the following treatment: 4-CPA, 4-CPA + WBPH, and control (Con).

Table S1. Verification of differently expressed genes by qRT-PCR.

Gene ID	Fold change (Transcriptome)	Fold change (qRT-PCR)
LOC_Os02g56120	22.80	14.35
LOC_Os01g09700	1.96	4.63
LOC_Os07g44440	119.74	125.78
LOC_Os01g27210	7.03	4.23
LOC_Os11g06770	3.69	6.92
LOC_Os05g35290	1.66	1.64
LOC_Os08g34790	1.91	3.87
LOC_Os09g34250	114.81	98.74
LOC_Os01g55940	203.93	189.56
LOC_Os01g45110	86.60	103.57
LOC_Os12g02400	0.68	0.63
LOC_Os01g58100	0.51	0.36
LOC_Os04g49210	0.68	0.51
LOC_Os11g02530	1.06	0.58
LOC_Os12g02450	0.69	0.26

Table S2. Selected genes and their primers used for qRT-PCR.

Gene ID	Definition	Primers	
LOC_Os02g56120	Auxin-responsive protein IAA	**F**	GGCGCCAACTACGTGAAGGT
		**R**	GCAGCGAGCTCGTCGTAGG
LOC_Os01g09700	1-aminocyclopropane-1-carboxylate synthase	**F**	CTCGAGGCCTACCTCCGTGA
		**R**	AACAGCGCGTTGTCCCTGAA
LOC_Os07g44440	Peroxidase	**F**	ACGTCGAGTCGCACAAGGAC
		**R**	CTCGTCGGCGTCGATCATGT
LOC_Os01g27210	Glutathione S-transferase	**F**	GCGGACCTCAGCCACTTCTC
		**R**	GCCATCACCTTCTGCACCGT
LOC_Os11g06770	Ethylene-responsive transcription factor ERF110	**F**	GCGGCGAGGAAGAAGAGGAG
		**R**	CGCGGACACCATCACAGACA
LOC_Os05g35290	Phenylalanine ammonia-lyase	**F**	CCTCGTCCCGCTCTCCTACA
		**R**	TGGGCTGCAGCTCGAAGAAG
LOC_Os08g34790	4-coumarate--CoA ligase	**F**	GCAGGGCCGGTGCTATCAAT
		**R**	ATCTTCAGCTCGGCGTTCCG
LOC_Os09g34250	UDP-glucosyl transferase	**F**	CGAGCTCCAGCCACAGGAAG
		**R**	GTCGTCGGCGAGAAGAGGTG
LOC_Os01g55940	Jasmonic acid-amido synthetase	**F**	CCGAGTGCGGGAAGGACAAG
		**R**	GGAGCCCACCGCTGTAGAAC
LOC_Os01g45110	Anthocyanin 3’-O-beta-glucosyltransferase	**F**	GGTCGCTCTTGCGAACAAGG
		**R**	GCCGAAGGAGACGTACACCA
LOC_Os12g02400	WRKY114	**F**	GCCCTCTCCGTCATGAACCA
		**R**	TTCCAGCGACGTGGTTGTCT
LOC_Os01g58100	Polyphenol oxidase	**F**	AACGAGCGATGTCGTGTTGC
		**R**	GTCAGAGTTTGGGCGCATGG
LOC_Os04g49210	Naringenin,2-oxoglutarate 3-dioxygenase	**F**	CGACTTCCTGCGCTTGCATT
		**R**	CGAGTAGGTGCCGACCACTT

LOC_Os11g02530	WRKY40	**F** AGGCAACAAAGACGGTGCAG
		**R** AGCAGGTATGCTGGCCGTAG
LOC_Os05g34030	Peptide transporter PTR2	**F** CGACGATCGGGTTCAATGCC
		**R** CCGACGATCGGTGTGAGGTT
LOC_Os12g02450	WRKY64	**F** GCAGAAGCACATCAACAACTCC
		**R** ACATGATCATCAGCTGATGCCG
LOC_Os03g50885	Actin	**F** TGGACAGGTTATCACCATTGGT
		**R** CCGCAGCTTCCATTCCTATG
